# Brief communication: patient satisfaction with the use of tablet computers: a pilot study in two outpatient home dialysis clinics

**DOI:** 10.1186/s40697-014-0022-9

**Published:** 2014-08-26

**Authors:** Kara Schick-Makaroff, Anita Molzahn

**Affiliations:** Faculty of Nursing, Level 3, Edmonton Clinic Health Academy, 11405-87 Ave, Edmonton, AB, T6G 1C9 Canada

**Keywords:** Tablet computer, iPad, Patient reported outcome measures, Electronic patient reported outcome measures, Health-related quality of life, Home dialysis, Nursing

## Abstract

**Background:**

Electronic capture of patients’ reports of their health is significant in clinical nephrology research because health-related quality of life (HRQOL) for patients with end-stage renal disease is compromised and assessment by patients of their HRQOL in practice is relatively uncommon.

**Objective:**

The purpose of this study was to evaluate patient satisfaction with and time involved in administering HRQOL and symptom assessment measures using tablet computers in two outpatient home dialysis clinics.

**Design:**

A cross-sectional observational study design was employed.

**Setting:**

The study was conducted in two home dialysis clinics.

**Patients:**

Fifty-six patients participated in the study; 35 males (63%) and 21 females (37%) with a mean age of 66 ± 12 (36-90 years old) were included. Forty-nine participants were on peritoneal dialysis (87%), 6 on home hemodialysis (11%), and 1 on nocturnal home hemodialysis (2%).

**Measurements:**

Measures included the Kidney Disease Quality of Life-36 (KDQOL-36), the Edmonton Symptom Assessment Scale (ESAS) and Participant’s Level of Satisfaction in Using a Tablet Computer.

**Methods:**

Using a tablet computer, participants completed the three measures. Descriptive statistics and bivariate correlations were calculated.

**Results:**

Participants’ satisfaction with use of the tablet computer was high; 66% were “very satisfied”, 7% “satisfied”, 2% “slightly satisfied”, and 18% “neutral”. On the 7-point Likert-type scale, the mean satisfaction score was 5.11 (SD = 1.6). Mean time to complete the measures was: Level of Satisfaction 1.15 minutes (SD = 0.41), ESAS 2.55 minutes (SD = 1.04), and KDQOL 9.56 minutes (SD = 2.03); the mean time to complete all three instruments was 13.19 minutes (SD = 2.42). There were no significant correlations between level of satisfaction and age, gender, HRQOL, time taken to complete surveys, computer experience, or comfort with technology. Comfort with technology and computer experience were highly correlated, *r* = .7, *p* (one-tailed) < 0.01.

**Limitations:**

Limitations include lack of generalizability because of a small self-selected sample of relatively healthy patients and a lack of psychometric testing on the measure of satisfaction.

**Conclusions:**

Participants were satisfied with the platform and the time involved for completion of instruments was modest. Routine use of HRQOL measures for clinical purposes may be facilitated through use of tablet computers.

## Abrégé

**Contexte:**

Étant donné la nécessité d’intégrer à la pratique les résultats rapportés par les patients la saisie électronique des rapports de patients au sujet de leur santé est importante. Elle l’est d’autant plus en recherche clinique en néphrologie, en raison du fait que la qualité de vie liée à la santé (QVLS) des patients atteints d’une néphropathie en phase terminale est compromise et que l’évaluation de la QVLS par les patients est relativement peu commune dans la pratique.

**Objectifs:**

L’objet de cette étude consistait à sonder la satisfaction des patients relativement à leur QVLS de même qu’au temps requis pour évaluer la QVLS et les symptômes, à l’aide de tablettes électroniques, dans deux cliniques externes de dialyse à domicile.

**Type d’étude:**

Une étude observationnelle transversale.

**Contexte:**

Cette étude a été menée dans deux cliniques de dialyse à domicile.

**Participants:**

Cinquante-six patients ont participé à cette étude.

dont 35 hommes (63%) et 21 femmes (37%) âgés en moyenne de 66 ± 12 (36 à 90 ans). Quarante-neuf participants étaient en dialyse péritonéale (87%), 6 étaient en hémodialyse à domicile (11%), et 1 était en hémodialyse nocturne à domicile (2%).

**Mesures:**

Les outils d’évaluation étudiés comprenaient la KDQOL-36 (qualité de vie des patients atteints de néphropathie-36) l’échelle d’évaluation des symptômes d’Edmonton (EESE), ainsi qu’une échelle de satisfaction des participants dans l’utilisation de tablettes électroniques.

**Méthodes:**

À l’aide de tablettes électroniques les participants ont effectué les trois mesures. On a calculé la statistique descriptive et les corrélations entre deux variables.

**Résultats:**

Le degré de satisfaction des participants quant à l’utilisation des tablettes électroniques était élevé

66% se sont dits « très satisfait » 7% « satisfait », 2% « plutôt satisfait », et 18% « neutre ». Sur une échelle de type Likert en 7 points, le degré de satisfaction moyen était de 5,11 (ÉT = 1,06). Le temps moyen requis pour effectuer les mesures était : Degré de satisfaction 1,15 minute (ÉT = 0,41), EESE 2,55 minutes (ÉT = 1,04), et KDQOL 9,56 minutes (ÉT = 2,03).

le temps moyen pour effectuer les trois mesures était : 13,19 minutes (ÉT = 2,42). Il n’existe aucune corrélation importante entre le degré de satisfaction et l’âge le sexe, la QVLS, le temps requis pour terminer les évaluations, l’expérience en informatique ou le degré de confort avec la technologie. Il existe une forte corrélation entre le degré de confort avec la technologie et l’expérience en informatique, r = 0,7, p (unilatéral) < 0,01.

**Limites de l’étude:**

Les limites comprennent un manque de généralisabilité en raison du petit volume de l’échantillon dont les participants étaient autosélectionnés parmi des patients relativement sains, de même que l’absence de tests psychométriques sur le degré de satisfaction.

**Conclusion:**

Les participants étaient satisfaits de la plateforme et du fait que le temps requis pour terminer les évaluations était modeste. Le recours régulier à des mesures de la QVLS à des fins cliniques pourrait être facilité par l’utilisation des tablettes électroniques. La facilité de collecte de données pourrait offrir des occasions de recourir aux données de QVLS dans le cadre des soins aux patients et dans les programmes d’amélioration de la qualité.

## What was known before

Despite the knowledge that patients living with end stage renal disease have compromised health related quality of life, measurement of this phenomenon in clinical practice is rare.

## What this adds

Health related quality of life measurement in clinical settings may be conducted expediently with the use of tablet computers. Participants’ overall satisfaction with the use of the tablet computers for completing the measures was generally high. Satisfaction was not related to age gender, time involved, experience, comfort, or health status.

## Background

End stage renal disease (ESRD) significantly influences patients’ perceptions of their health and the quality of their lives. Health-related quality of life (HRQOL) is subjective and it can only be accurately assessed by the patient [1]. Although there is ample research identifying that HRQOL is compromised for patients living with ESRD [2,3], measurement of HRQOL in clinical practice is relatively uncommon [4]. Use of computer-based technologies may facilitate use of these measures in practice, if they are acceptable to patients. Hence, the aim of this study was to assess patient satisfaction with use of the tablet computer for completion of HRQOL and symptom assessment measures in outpatient home hemodialysis and peritoneal dialysis clinics and to ascertain the time required to complete these measures. The research questions were: How satisfied are outpatient home hemodialysis and peritoneal dialysis patients with use of tablet computers to score HRQOL and symptom assessment measures? How long did it take for participants to complete the measures using tablet computers?

Recently, in the United States, the Centers for Medicare and Medicaid Services (CMS) [5] mandated that every dialysis unit must routinely measure HRQOL of patients at least annually as a prerequisite for coverage. The rationale for this CMS requirement was based in part on the finding that lower HRQOL scores are good predictors of mortality and hospitalization [6,7].

There is some recognition that HRQOL data could be useful to practitioners. Researchers have found that use of these data had a positive impact on communication between physicians and patients (without prolonging interactions) [8], helped physicians to identify symptoms that otherwise might have been unnoticed [9], and impacted healthcare professionals when patient reported outcome (PROs) were used as a management tool in an outpatient setting with a specific patient population [10]. However, use of HRQOL and other PROs has been limited because paper and pencil administration precludes rapid scoring and presentation of results. Routine HRQOL assessment, with the use of touch-screen computers, has been found to significantly improve nurses’ awareness of patients’ experiences of pain, level of functioning, and overall quality of life [11], and increase communication and management of patients, without prolonging the length of patient visits [12].

Researchers cannot necessarily assume that translations of PROs from paper to electronic versions are equivalent [13–15]. The need to test measurement equivalence is determined by the level of modification required. Usability testing encompasses formal documentation of respondents’ ability to use the chosen electronic platform, follow instructions, and answer the questions [13].

## Methods

### Study design

We employed a cross-sectional observational study design. We attended every home dialysis clinic held two/three times a week (in one center) or every second week (in the other center) over a three-month time period in two cities on the west coast of Canada. When patients came for their regularly scheduled clinic appointment, they were invited by a third party to participate in the study. If the person was interested, they were directed to approach the researcher who was seated in the waiting room area. Over the three-month period, 83 patients attended the two outpatient clinics, and 56 patients chose to participate (68% response rate). Typically, patients attend an outpatient clinic every three months, so all home dialysis patients in these two cities were screened for participation during this period of time.

### Participants

Patients were invited to participate if they were on dialysis at home (either peritoneal or hemodialysis), over 19 years of age, and were willing to be enrolled in the study. Exclusion criteria included: mild or severe cognitive impairment, lack of English language proficiency, inability to read, and attendance at the clinic for a medical crisis. There was no requirement for patients to be knowledgeable regarding use of a tablet computer; the researcher provided a demonstration and was available to assist if needed. We attained ethics approval from the University of Alberta and the Vancouver Island Health Authority. Signed informed consent forms were obtained from all participants.

### Instruments

Three survey instruments were used to collect data. The first was the Edmonton Symptom Assessment Scale (ESAS) [16], modified for renal patients [17,18]. The second instrument was the KDQOL-36 developed by Hayes et al. [19]. The third instrument, Participant’s Level of Satisfaction in Using a Tablet Computer, was developed for the purposes of the study. It includes three items measured on a 7-point Likert-type scale. The instrument included the following questions: How satisfied were you with completing the two surveys on a tablet computer/portable device? How much computer experience do you have? How comfortable are you in using computer technology generally? Satisfaction was our focus because end-user satisfaction is the most frequently used measure of Information System/Information Technology success [20].

### Tablet technology

The tablet computer used for this study was the iPad™ (iPad is a trademark of Apple, Inc). The ESAS and Participant’s Level of Satisfaction questionnaires were designed as a newly created application to be run on the FileMaker Go iPad app using iOS 7. The computer programmer constructed the application using FileMaker 12. It was designed for older adults with minimal computer experience. The app used a large font (32 point for the question, 36 point for the response), black writing on a white background, no graphics, and simple directions.

The KDQOL-36 was completed online through *KDQOL Complete* offered by Medical Education Institute, a non-profit organization devoted to helping people with chronic kidney disease manage and improve their own health [21]. *KDQOL Complete* is available for use in dialysis settings. It offers online scoring in real time^a^.

In this study, only minor modifications to the instruments were made for the electronic platform [13]. Specifically, respondents touched the screen instead of circling a number, and the number of items seen at one time on a screen was different compared to the number of items seen on a piece of paper. Satisfaction with use of the technology was assessed with the Participant’s Level of Satisfaction questionnaire. Time stamps were utilized in the tablet application and on the KDQOL Complete to track how long it took participants to complete each survey.

For this study, direct access to the health authority’s internet was not allowed. As a result, tablet computers with cellular capacity were used, and data collection for the KDQOL-36 proceeded using a 3G connection. On the two occasions that the 3G connection was dropped, a paper copy of the KDQOL-36 was used in the interim until connection was restored. Completion of the ESAS and Participant’s Level of Satisfaction questionnaires using the FileMaker Go tablet app did not require an internet connection. Data from the application was downloaded to a secure data repository.

### Data analysis

Descriptive statistics (means, standard deviation) were calculated for each item. Spearman’s correlation coefficient were used to assess relationships between pairs of variables. SPSS (version 21) was used.

## Results

Characteristics of participants are presented in Table 1. The mean age was 66 years with a range from 38 to 91 years. Notably, only 2 out of 56 participants (4%) had previously used a tablet computer.

**Table 1:** **Characteristics of participants**

**Clinical characteristics**	
Gender (M/F)	35 (63%)/21 (37%)
Age, years	66 ± 12
Diabetes	16 (29%)
**Race**	
Caucasian	51 (91%)
First Nations	2 (3.5%)
Asian	1 (2%)
Hispanic	2 (3.5%)
**Employment status**	
Retired due to age/preference	39 (70%)
Retired (disability)	7 (12%)
Medical leave of absence	6 (11%)
Full-time employee	3 (5%)
Homemaker	1 (2%)
**Dialysis modality**	
Peritoneal dialysis	49 (87%)
Home hemodialysis	6 (11%)
Nocturnal home hemodialysis	1 (2%)
Computer experience*	
None at all	8 (15%)
A little bit	9 (17%)
A little	8 (15%)
Average	15 (28%)
A little above average	3 (5%)
A lot	10 (18%)
An exceptional amount	1 (2%)
Comfort with computer technology*	
Very uncomfortable	5 (9%)
Uncomfortable	7 (13%)
Fair	16 (30%)
Good	12 (22%)
Very comfortable	11 (20%)
Excellent	3 (6%)

Values are n (%) or mean ± SD.

*Responses are missing for two participants.

Participant satisfaction was high with 66% reporting that they were “very satisfied”, 7% “satisfied”, 2% “slightly satisfied”, and 18% “neutral”; 3.6% reported dissatisfaction. The mean satisfaction score was 5.11 (SD = 1.6) on the 7-point scale. Again, using a 7-point scale, the mean computer experience score was 2.56 (SD = 1.7), and comfort using computer technology was 2.48 (SD = 1.4). A total of 75% of participants reported they had between no computer experience and average experience, yet 78% said they had fair to excellent comfort with computer technology.

Bivariate correlations were examined between satisfaction and age, gender, time, experience, comfort, and the global health item from the KDQOL-36. Satisfaction with use of the tablet computers was not correlated with either experience or comfort. We hypothesized that more computer experience would be correlated with greater comfort with technology; a one-tailed Spearman’s correlation coefficient between comfort and experience was 0.7, which was statistically significant (p < 0.01).

The mean length of time to complete the questionnaires is reported in Table 2. Of the 56 participants, all but two of them completed all three surveys. As noted in Table 2, there were a number of missing or incorrect time stamps. The reason for this was that participants frequently did not complete a survey at one continuous point in time because of appointments with a healthcare professional during their visit to the outpatient clinic.

**Table 2:** **Time (in Minutes) taken to complete surveys**

**Measure**	**Mean**	**SD**	**Range (in minutes)**	***n***
Level of Satisfaction (3)	1.15	0.41	1-3	48
ESAS (11)	2.55	1.04	1-6	42
KDQOL-36	9.56	2.03	6-13	27
**Total time for all 3 surveys**	13.19	2.42	8-18	21

## Discussion

Participants’ overall satisfaction with use of the tablet computer for completing the instruments was generally high and it seems that satisfaction is not related to age, gender, time involved, experience, comfort, and health status. Perhaps other contextual factors, such as design of the app, and interviewer instructions and support may have influenced their satisfaction. Because only 4% of the sample had ever used a tablet computer previously, initial instruction was required to use the device and respond to questions. It is likely that less instruction will be required for administration of the questionnaires using this platform at future visits. We anticipate that in the future, as electronic devices become more common and as baby-boomers enter retirement, less education/support for the use of tablet computers will be required for patients to use them in clinical settings.

Confirming our findings, Chang et al. [15] also concluded that age and previous computer experience did not explain acceptance of an electronic platform. Pouwer et al. [22] also found that e-PROs were very easy for research participants to use even if they had little computer experience.

Outpatient clinic settings are considered to be ideal for use of PRO feedback as a management tool with particular populations, such as ESRD; the course of action with targeted solutions may be clearer to practitioners, and there may be greater room for improvement of outcomes and reports of HRQOL with patients who present with advanced symptoms [10,23].

Despite having a 68% response rate, the study was limited due to its small sample size. Data were collected from self-selected patients using a home dialysis modality and they were not representative of the larger ESRD population. Further, patients who were experiencing medical crises were not included, so healthier ESRD patients were overrepresented. The study was also limited in that it only included participants from one geographic area on the west coast of Canada. Another limitation is the lack of psychometric testing for the measure of satisfaction, which was developed for the purposes of the study and modeled on similar measures. Another concern was that in our study, 3.6% of participants reported that they were “very dissatisfied” with using the tablet. However, in the interviews that followed survey completion, these same participants said that they enjoyed using the technology, suggesting that they may have made errors in the rating. This error rate may be an area for future research pertaining to the use e-PROs.

## Conclusion

We found that patient satisfaction was high with completion of HRQOL and symptom assessment measures on tablet computers. The time required to complete the surveys was modest. As electronic capture of patient report data becomes routine in healthcare settings, use of HRQOL assessment for clinical purposes may be facilitated through use of tablet computers. This is important both for practice and research settings where PRO results have the potential to be analyzed in real-time to facilitate patient management and to enhance communication between patients and healthcare providers [8–12,15]. The ease of electronic capture of PROs may offer opportunities to use HRQOL data for patient care and/or quality improvement programs.

## Endnote

^a^The KDQOL Complete database is housed on a server in the United States, which means that under the US Patriot Act, there is a possibility that information about the participant could be accessed without their knowledge or consent by the US government. To preserve confidentiality of data, participants were only identified by their assigned ID codes on the KDQOL Complete website. Participants were also informed of this on the consent form.

## Abbreviations

CMS: Centers for medicare and medicaid services
e-PRO: Electronic patient reported outcome
ESAS: Edmonton symptom assessment scale
ESRD: End stage renal disease
HRQOL: Health-related quality of life
KDQOL-36: Kidney disease quality of life
PRO: Patient reported outcome
